# Unveiling the nanotoxicological aspects of Se nanomaterials differing in size and morphology

**DOI:** 10.1016/j.bioactmat.2022.06.014

**Published:** 2022-06-25

**Authors:** Hana Stepankova, Hana Michalkova, Zbynek Splichal, Lukas Richtera, Pavel Svec, Tomas Vaculovic, Jan Pribyl, Martin Kormunda, Simona Rex, Vojtech Adam, Zbynek Heger

**Affiliations:** a Department of Chemistry and Biochemistry, Mendel University in Brno, Zemedelska 1, Brno, CZ-613 00, Czech Republic; b Central European Institute of Technology, Brno University of Technology, Purkynova 123, Brno, CZ-612 00, Czech Republic; c Department of Chemistry, Masaryk University, Kamenice 5, Brno, CZ-625 00, Czech Republic; d Institute of Laboratory Research on Geomaterials, Faculty of Natural Sciences, Comenius University in Bratislava, Mlynska dolina, Ilkovicova 6, 842 15 Bratislava, Slovak Republic; e Central European Institute of Technology, Masaryk University, Kamenice 5, Brno, CZ-625 00, Czech Republic; f Department of Physics, Faculty of Science, J. E. Purkyne University, Pasteurova 1, Usti nad Labem, CZ-400 96, Czech Republic

**Keywords:** Aspect ratio, Biocompatibility, Nanotoxicology, Nanorods

## Abstract

Although the general concept of nanotechnology relies on exploitation of size-dependent properties of nanoscaled materials, the relation between the size/morphology of nanoparticles with their biological activity remains not well understood. Therefore, we aimed at investigating the biological activity of Se nanoparticles, one of the most promising candidates of nanomaterials for biomedicine, possessing the same crystal structure, but differing in morphology (nanorods *vs.* spherical particles) and aspect ratios (AR, 11.5 *vs.* 22.3 *vs.* 1.0) in human cells and BALB/c mice. Herein, we report that in case of nanorod-shaped Se nanomaterials, AR is a critical factor describing their cytotoxicity and biocompatibility. However, spherical nanoparticles (AR 1.0) do not fit this statement and exhibit markedly higher cytotoxicity than lower-AR Se nanorods. Beside of cytotoxicity, we also show that morphology and size substantially affect the uptake and intracellular fate of Se nanomaterials. In line with *in vitro* data, *in vivo* i.v. administration of Se nanomaterials revealed the highest toxicity for higher-AR nanorods followed by spherical nanoparticles and lower-AR nanorods. Moreover, we revealed that Se nanomaterials are able to alter intracellular redox homeostasis, and affect the acidic intracellular vesicles and cytoskeletal architecture in a size- and morphology-dependent manner. Although the tested nanoparticles were produced from the similar sources, their behavior differs markedly, since each type is promising for several various application scenarios, and the presented testing protocol could serve as a concept standardizing the biological relevance of the size and morphology of the various types of nanomaterials and nanoparticles.

## Highlights

• Three types of Se nanomaterials are synthesized with the same crystallinity.
• Se nanomaterials differ in size and morphology.
• Size and morphology markedly affects uptake and intracellular fate *in vitro*.
• Aspect ratio is a critical factor predetermining cytotoxicity of Se nanorods.
• High aspect ratio of nanorods is linked with serious kidney tissue damage.

## Graphical abstract

**Figure:**
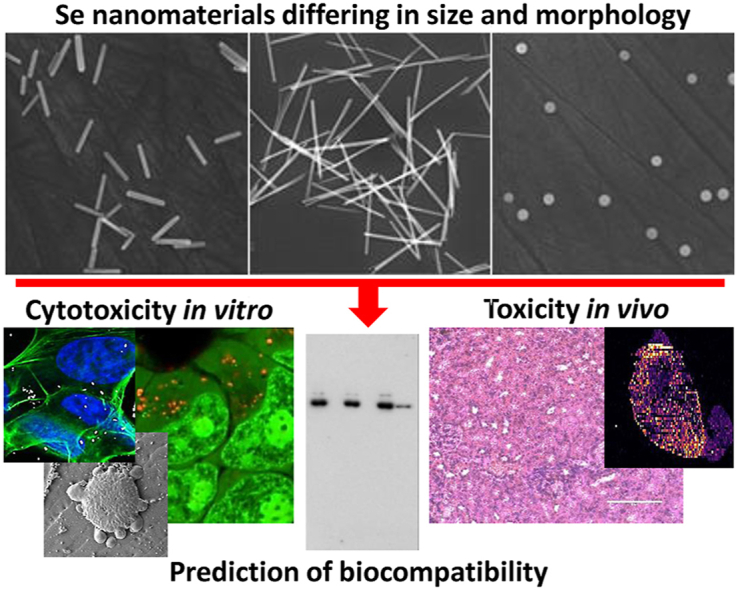

## 1 Introduction

Over the past several decades, the society has witnessed rapid advances in the field of nanotechnology resulting in the production of a vast number of engineered nanomaterials with various outstanding properties attributed to their size, large surface area and concomitant surface display of their constituent atoms [1]. The size of nanomaterials is resembling that of biomolecules and their structures; hence, these nanomaterials can be employed for various systems and environment, which led to advancement in variety of fields including biology, medicine, biotechnology and healthcare [2]. Nanomaterials also play important role in the agro-food-feed sectors, such as crop production, nutrition, enhancement of water quality, food packaging, *etc*. [3].

Recent advances in nanoengineering of materials have hand-in-hand led to urgent need of detailed understanding of the relations between size and morphology of nanomaterials with their nanotoxicological profiles, completely differing from those of former bulk materials [4]. As we showed in our previous study on nanotoxicology of Pt-based nanoparticles, in the nanoscaled world even the smallest size differences can have crucial biological effects [5]. In line with this statement, a number of reports have demonstrated that smaller spherical nanoparticles are more cytotoxic than their larger counterparts [[5], [6], [7], [8], [9]]. In the case of 1-D nanomaterials, aspect ratio (AR) is considered to be a major factor predeterming the nanotoxicity of materials and several studies have demonstrated that higher AR associates with higher toxicity [[10], [11], [12], [13]]. However, it has to be noted that a number of contradictory data can be found, some of them showing no size-dependent toxicity, lower toxicity of smaller nanoparticles or a reverse correlation between the toxicity and AR [[14], [15], [16]]. These contradictions are often accompanied by heterogeneity in the synthetic routes, characterization and even testing protocols. Considering these facts, the consensus on relation between size- and morphology with nanotoxicity cannot be unequivocally established, and each nanomaterial must be comprehensively examined with respect to the target application.

Among the most promising nanomaterials, Se nanoparticles are widely accepted in biomedicine and food science. Se is a crucial factor of various selenoproteins acting as antioxidants, and is also essential for maintenance of human health [17]. Hence, it is not surprising that Se nanomaterials have been tested in a plethora of applications including anticancer, antimicrobial and anti-inflammatory experiments, food supplementation, and many others [[18], [19], [20], [21]].

Since 2001, the number of papers dealing with any aspect of Se nanomaterials has rapidly grown from few to more than 4800 papers in total published in 2019 (according to the Web of Science Core Collection). From this number, 822 papers dealt with any aspect of Se nanomaterials and their toxicity in distinct model organisms (Supplementary Fig. 1). Although Se nanomaterials are considered biocompatible, the available literature is inconsistent in this fundamental question. Indeed, the contradictions are plausibly highly multifactorial and could be related to various aspects such as surface chemistry, charge, enzymomimetic activity, morphology or size. Noteworthy, the papers focusing on size-dependent toxicity are still very rare (only 8 records), and most of them aimed on various synthesis routes providing only limited insights into the biological effects of Se nanomaterials [12,22,23]. In addition, to the best of our knowledge, no investigation has been done to elucidate the morphology-dependent toxicity of Se nanomaterials.

Hence, the aim of this work was to generate a comprehensive and homogenous set of data on biocompatiblity and toxicity of Se nanomaterials of the same crystal structure, but different morphologies (spherical *vs*. nanorods, NR) and sizes [comparable in one dimension (38 ± 4 – 45 ± 4 nm) but varying in AR (1.0 – ∼22.3)]. We found that AR of nanorods positively correlated with toxicity at the *in vitr*o and *in vivo* level. Interestingly, higher-AR SeNR-2 exhibited even more pronounced toxic effects than spherical Se nanoparticles (SeNPs). Furthermore, it was revealed that Se nanomaterials were able to alter intracellular redox homeostasis, and in a size- and morphology-dependent manner affected the acidic intracellular vesicles and cytoskeletal architecture. For the first time, we provide a comprehensive evaluation of size- and morphology-dependent nanotoxicological aspects of Se nanomaterials. Importantly, the presented data could also contribute to a broad spectrum of future biomedical applications.

## 2 Materials and methods

### 2.1 Chemicals

All chemicals in this study, unless otherwise stated, were purchased from Sigma-Aldrich (St. Louis, MO, USA). The demineralized water was produced using reverse osmosis apparatus Aqual 25 (Aqua Osmotic, Tisnov, Czech Republic). The demineralized water was further treated with the Milli-Q Direct QUV equipment furnished with UV lamp (Aqua Osmotic, Tisnov, Czech Republic). The resistance value was 18.20 MΩ cm (at 25 °C).

### 2.2 Synthesis of Se nanomaterials

Spherical SeNPs were synthesized according to our previously published protocol [21]. The synthesis of nanorod-shaped Se nanomaterials was carried out in a chemical reduction process previously described by Chiou and coworkers [24]. In the typical procedure, the sodium salt of carboxymethyl cellulose (Na-CMC, MW 90,000 Da, 4.0 wt%) was dissolved in Milli-Q water (9.0 mL) at room temperature, followed by addition of NaOH (1.0 mL, 1.0 M) and selenous acid (1.0 mL, 1.0 M). The mixed solution was then stirred vigorously at room temperature until it became transparent, producing the Na-CMC-stabilized H_2_SeO_3_ solution. Subsequently, fresh Na[BH_4_] (1.0 mL, 1.0 M) was added dropwise (during 60 s for SeNR-1 and 2 s for SeNR-2) to the Na-CMC-stabilized H_2_SeO_3_ to carry out the reduction reaction. After stirring at room temperature (2 h), brown suspending solids were produced. The precipitate was collected by centrifugation at 10,000×*g* for 10 min and washed three times with Milli-Q water and two times with ethanol. Before biological experiments, Se nanomaterials were evaluated for endotoxin content using Pierce™ LAL Chromogenic Endotoxin Quantitation Kit (Thermo Fisher Scientific, Waltham, MA, USA).

### 2.3 Physico-chemical characterization of Se nanomaterials

Morphology of Se nanomaterials was examined using field-emission scanning electron microscopy (FE-SEM) MAIA 3 (Tescan, Brno, Czech Republic). FE-SEM micrographs were obtained in ultra-high resolution mode using the In-Lens SE detector at working distance set between 2.94 and 5.02 mm at 5 kV acceleration voltage. The atomic force microscopy (AFM) analyses of Se nanomaterials were performed on nanomaterials fixed on mica sheets using Bruker Dimension FastScan (Bruker Nano Surface, Santa Barbara, CA, USA) operated in tapping mode. Parameters of visualization were as follows: set point value 3.5 nm, iGain 0.8, PGain 5.5, piezo Z scale range 500 nm. For AFM data post processing and for graphical output, Gwyddion software version 2.51 was used [25]. Hydrodynamic diameter (HDD), ζ-potential and polydispersity index (PDI) were investigated using Zetasizer Nano ZS (Malvern Instruments, Malvern, UK). The instrument setup was as follows: temperature 25 °C, measurement angle 173° backscatter, adsorption coefficient 10^−3^ and equilibration time 120 s. In all analyses, refractive index of dispersive phase and dispersive environment was set to 2.790 and 1.333, respectively. Surface chemistry of Se nanomaterials was investigated using X-ray photoelectron spectroscopy (XPS) using Axis Supra with monochromatic Al Kα X-ray radiation, emission current of 15 mA and hybrid lens mode (Kratos Analytical, Manchester, UK). High resolution spectra were recorded with pass energy of 20 eV. XPS spectra were analyzed using CasaXPS software version 2.3.22. Spectra were calibrated using C–C component of C 1s peaks at 284.50 eV. The Shirley algorithm was used to establish the background of the spectra and the Gaussian–Lorentzian line shape was used to fit the XPS peaks. Se nanomaterials were further characterized using energy-dispersive X-ray fluorescence (XRF), organic elements composition analysis, Raman spectrometry and Fourier-transform infrared spectroscopy (FT-IR) with a detailed methodological description provided in Supplementary information.

### 2.4 Analysis of dissolution of Se nanomaterials

To evaluate the dissolution of Se nanomaterials in Milli-Q water and cell culture medium (DMEM), 50 μg/mL of each Se nanomaterial was aseptically incubated for 24 or 48 h at 37 °C. After the incubation, dissolved Se was separated from nanoparticles by ultracentrifugation at 450,000×*g* for 30 min (Himac CS150NX, Hitachi, Tokyo, Japan). After acidic microwave digestion (Anton Paar GmbH, Graz, Austria), dissolved Se was quantified from supernatant using inductively coupled plasma mass spectrometry (ICP-MS) (7700x ICP-MS, Agilent Technologies, Santa Clara, CA, USA). The results were normalized to the amount of detected fully soluble Se ICP standard (50 μg/mL, Merck Millipore, Molsheim, France).

### 2.5 Cell lines and cell culturing

Two human cell lines were used: *i*) the HBL-100 cell line, which is non-malignant epithelial cell line established from the milk of a nursing woman three days post-delivery and *ii*) the SH-SY5Y cell line, which is a neuroblast-like cell line obtained as a third successive sub-clone of the SK-N-SH cell line, originally established from a bone marrow biopsy of a metastatic neuroblastoma. HBL-100 cells were cultured in DMEM, SH-SY5Y cell were cultured in IMDM, both supplemented with 10% fetal bovine serum (FBS), penicillin (100 U/mL) and streptomycin (0.1 mg/mL). Prior each analysis, the cells were counted using Countess II FL (Thermo Fisher Scientific).

### 2.6 Evaluation of cytotoxicity of Se nanomaterials

Cytotoxicity of Se nanomaterials was investigated using the XTT assay (2,3-bis-(2-methoxy-4-nitro-5-sulfophenyl)-2*H*-tetrazolium-5-carb-ax-anilide). To exclude a false positivity due to possible inherent enzymomimetic activity of Se nanomaterials, the obtained results were validated by Trypan blue exclusion according to our previously published study [21]. To investigate the effect of Se nanomaterials on clonal efficiency in terms of formation of progeny colonies, the cells were seeded in a 6-well plate at a density of ∼1 × 10^3^ cells per well and incubated overnight. Then, the cells were treated with Se nanomaterials (24hIC_50_ concentrations) for 24 h. After medium renewal, the cells were incubated for 8 days. Then, the wells were washed with phosphate-buffered saline (PBS, pH 7.4) and fixed with methanol:acetic acid solution (3:1, *v/v*) for 5 min. After crystal violet staining, the colonies were analyzed using EVOS FL Auto Cell Imaging System (Thermo Fisher Scientific). The clonal efficiency was calculated as the number of colonies containing at least 50 cells. The antimigratory properties of Se nanomaterials were explored through wound-healing assay. For this purpose, suspension of ∼1 × 10^6^ cells/well was seeded to 6-well plates and incubated overnight. The cell monolayer was then scraped with a p20 pipet tip to create a cell-free zone. After washing with PBS, the cells were administered with 24hIC_25_ concentrations of Se nanomaterials. The micrographs of wound closing were captured at regular time points (up to 72 h) under phase contrast microscope. The migration rate (free wound area) was calculated from 6 independent spots in each well.

### 2.7 Confocal reflectance microscopy (CRM) and analysis of F-actin cytoskeletal network

After seeding onto coverslips (∼1 × 10^5^ cells/coverslip) the cells were incubated overnight. Then, the cells were administered with Se nanomaterials (24hIC_50_, 3 h), washed with PBS and fixed in 4% formaldehyde (20 min, 25 °C). After permeabilization (0.2% Triton X-100) and 3 × washing with PBS, the cells were blocked with 3% bovine serum albumin and stained using Alexa Fluor 488-phalloidin to enable visualization of F-actin architecture. After final rinsing with PBS, the coverslips were mounted with ProLong™ Gold Antifade Mountant containing 4′,6-diamidino-2-phenylindole (DAPI). The cells were visualized in CRM mode using LSM 880 (Carl Zeiss, Jena, Germany) by collecting the light backscattered from the Se nanomaterials upon irradiation by an argon 514 nm laser. Quantitation of the number of reflection spots was carried out using the ImageJ software package (National Institute of Health, Bethesda, MA, USA) using the “analyze particles” function. The threshold was optimized to the values in which the background signals were eliminated and only Se nanomaterials were observable. Morphometric evaluation of F-actin cytoskeleton alterations due to Se nanomaterials exposure was performed from the micrographs acquired in the AiryScan superresolution mode on the LSM 880 (Carl Zeiss). The coherency and density of F-actin fibers were quantified using the ImageJ (National Institute of Health) according to Clemons et al. [26].

### 2.8 Evaluation of effect of Se nanomaterials on cell morphology

The cellular morphology was explored using cryo-FE-SEM. The cells were seeded onto carbon stubs (∼1 × 10^4^ cells/stub) and incubated overnight. Then, the cells were treated with Se nanomaterials (24hIC_50_) and after 24 h incubation; the cells were frozen in liquid nitrogen in PP3010 Cryo-SEM Preparation System (Quorum Technologies, Sussex, UK). The cellular morphology was investigated under high vacuum using MAIA3 FE-SEM (Tescan). Micrographs were obtained using the ET SE detector at working distance between 2.22 and 3.20 mm at 1 kV acceleration voltage.

### 2.9 Lipid peroxidation imaging and quantitation of reactive oxygen species (ROS)

For lipid peroxidation imaging, the cells were seeded in 6-well plate (∼1 × 10^6^ cells/well). After overnight incubation, the cells were treated with Se nanomaterials (24hIC_50_) for 12 h. The lipid peroxidation was examined after staining with Image IT™ Lipid Peroxidation Kit (Thermo Fisher Scientific) utilizing cumenehydroperoxide (CHP) as a positive control. The cells were observed under EVOS FL Auto Cell Imaging System (Thermo Fisher Scientific). 590/510 nm ratio was calculated using the Simple PCI (Hamamatsu, Sewickley, PA, USA). Intracellular ROS were quantified in 10^4^ events on BD Accuri C6 Plus flow cytometer (BD Biosciences, Franklin Lakes, NJ, USA) after, treatment of the cells with Se nanomaterials (24hIC_50_, 12 h) and staining with CellROX® Green reagent according to the manufacturer's instructions.

### 2.10 Plasmid DNA cleavage assay

The capability of Se nanomaterials to cause oxidative damage to plasmid DNA (pDNA) was examined through their ability to cause a conversion of mostly supercoiled pDNA (pX330-U6) to nicked and linear forms. The pDNA (50 ng/μL) in 50 mM Tris-HCl buffer with 50 mM NaCl was treated with the indicated concentrations of Se nanomaterials for 1 h (37 °C). After incubation, the samples were resolved on 1% neutral agarose gel with 40 mM Tris-acetate, 1 mM EDTA and 0.5 μg/mL of ethidium bromide (EtBr). Bands were visualized using Azure c600 (Azure Biosystems, Dublin, CA, USA).

### 2.11 Acridine orange (AO) relocation assay for lysosomal staining

Staining of lysosomes and lysosomal membrane destabilization (LMD) was carried out using metachromic fluorophore AO according to Zareba et al. [27]. For the assays, cells (∼1 × 10^5^ cells) were grown in cell culture dishes. Then, the cells were loaded with AO (5 μg/mL) for 15 min, rinsed with culture media and incubated with 24hIC_50_ concentrations of Se nanomaterials. After 4 h incubation, the culture medium was removed and the cell were rinsed with PBS (3 × ). Then, the cells were observed under LSM 880 (Carl Zeiss). The fluorescence of AO in cells was also analyzed using Infinite 200 PRO plate reader (Tecan, Maennedorf, Switzerland) at *λ*_em_ 485 nm and *λ*_exc_ 530 nm (green cytoplasmic AO) and *λ*_exc_ 620 nm (red lysosomal AO).

### 2.12 Interactions between blood circulation environment and Se nanomaterials *in vitro*

Hemocompatibility of Se nanomaterials was examined using human red blood cells (RBCs) (Zen-Bio, Durham, NC, USA) according to the previously published protocol [5]. Degree of hemolysis was calculated as follows: %*hemolysis* = [(A_*t*_ − A_*c*_)/(A_*100*_% − A_*c*_)] × *100*, where A_*t*_ stands for the absorbance of supernatant from samples; A_*c*_ is the absorbance of the supernatant from negative control (PBS, pH 7.4); and A_*100*%_ is the absorbance of the supernatant from positive control (0.1% Triton X-100), causing complete lysis of RBCs. Furthermore, protein corona formation and opsonization with C3 was investigated using commercially available human serum following previously published workflow [28].

### 2.13 Murine model and treatment protocol

Twenty six-week-old female BALB/c mice were used for the *in vivo* study (five per each experimental group). All animals were housed in individually ventilated cages at a 12/12 h light/dark cycle and were provided *ad libitum* with standard diet and water. The use of the animals followed the European Community Guidelines as accepted principles for the use of experimental animals. The animals were administered with a single-dose application of Se nanomaterials (150 μg/mL in 100 μL of sterile PBS) into the tail vein. During the experiments, the animals were weighted on a regular basis. After eight days post administration, the mice were euthanized by overdosing with Narkamon/Rometar and blood and selected organs were collected and immediately processed. Part of the whole blood was separated by centrifugation to obtain platelet-rich plasma (PRP) and plasma. Organs were either homogenized or prepared as formalin-fixed paraffin-embedded (FFPE) blocks, which were subsequently cut to 8 μm sections using sliding microtome Leica SM2010 R (Leica, Wetzlar, Germany). Whole blood, PRP, plasma and organs were analyzed for Se content and spatial distribution in tissue slices as described below.

### 2.14 Quantitation of amount of Se in homogenates

Homogenates of liver, kidney, heart, plasma, blood, and spleen were spotted on the membrane. The spots were analyzed by laser ablation with ICP-MS (LA-ICP-MS). The LA-ICP-MS setup consisted of a laser ablation system UP213 (New Wave Research, Fremont, USA) emitting laser radiation of 213 nm with a pulse duration of 4.2 ns. Ablated material was carried out from the ablation cell by helium flow (1.0 L/min). Before entering into ICP-MS Agilent 7500ce (Agilent Technologies) with quadrupole analyser, the helium was mixed with argon (0.6 L/min). The ablation was done using two line scans on each spot under optimized ablation parameters (laser beam fluence 2.3 J/cm^2^, repetition rate 20 Hz, laser beam diameter 110 μm, scan speed 220 μm/s). Isotope ^77^Se was used for monitoring of Se amount. The amount of Se is expressed as the background-corrected intensity of ^77^Se averaged across two line scans on each sample.

### 2.15 Spatial distribution of Se in kidney sections

Spatial distribution of Se in kidney sections was determined by LA-ICP-MS. The system consisted of a laser ablation system Analyte G2+ (Teledyne, El Segundo, CA, USA) emitting laser radiation of 213 nm and equipped with two-volume ablation cell. Ablated material was carried out from the cell by helium flow with flow rates of 0.6 and 0.3 L/min. Before entering into ICP-MS Agilent 7900 (Agilent Technologies) with quadrupole analyser, the helium was mixed with argon (0.6 L/min). Imaging of elements was done under following ablation parameters: laser beam fluence 3.0 J/cm^2^, repetition rate 20 Hz, laser beam diameter 50 μm, scan speed 100 μm/s, the distance between lines 50 μm. Data processing was done by lab-made software ILAPS.

### 2.16 Kidney histopathology

The FFPE kidney sections were mounted on glass slides, deparaffinized and stained with haematoxylin-eosin (H&E). The mounted specimens were observed and scored under light microscopy (EVOS FL Auto Cell Imaging System, Thermo Fisher Scientific). For a semi-quantitative comparison of the structural changes, the abnormalities in the tissue sections were graded from 0 (normal structure) to 3 (severe pathological changes).

### 2.17 Descriptive statistics

For the statistical evaluation of the results, the mean was taken as the measurement of the main tendency, while positive and negative error was taken as the dispersion measurement. Differences were analyzed using paired (to analyze the effect of individual Se nanomaterial in different conditions) or unpaired *t*-test. For analyses, Software Statistica 12 (StatSoft, Tulsa, OK, USA) was employed.

## 3 Results

### 3.1 Physico-chemical characterization of Se nanomaterials

**Fig. 1:**
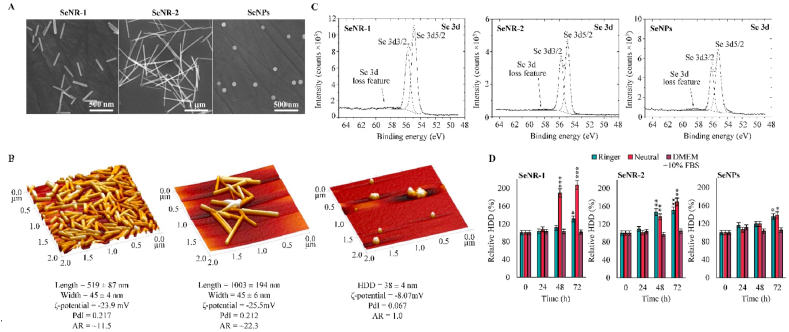
Physico-chemical properties of Se nanomaterials. (A) FE-SEM and (B) 3D AFM micrographs showing morphology of tested Se nanomaterials. Values below describe size, ζ-potential, polydispersity index (PdI) and AR. (C) XPS spectra showing Se 3d peaks of synthesized Se nanomaterials. (D) Temporal evolution of HDDs of Se nanomaterials incubated in solutions mimicking distinct physiological environments and cell culture conditions. The data represent three separate experiments (*n* = 3) and are expressed as mean values ± SD. *p < 0.05, **p < 0.01, ***p < 0.005 related to the initial time-point (0 h).

**Fig. 2:**
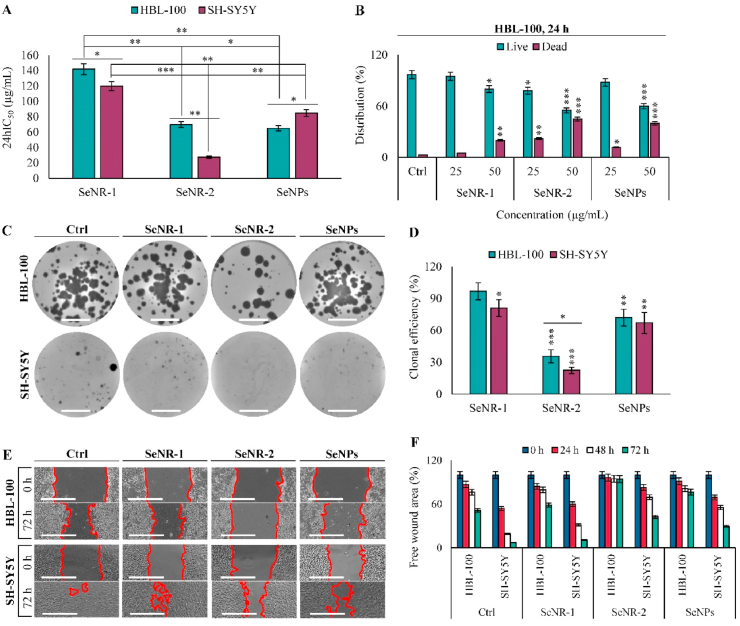
Cytotoxic activity and antiproliferative activity of Se nanomaterials. (A) 24hIC_50_ values in HBL-100 and SH-SY5Y cells obtained by MTT assay. (B) Validation of cytotoxic activity of Se nanomaterials in HBL-100 cells analyzed by trypan blue exclusion. (C) Representative cell culture dishes stained with crystal violet at the end-point of the colony formation assay. Scale bar, 10 mm. (D) Quantitation of clonal efficiency presented as percentage of colonies in the comparison to untreated cells used as a control. (E) Representative micrographs showing healing of an artificial gap in cellular monolayer after treatment with Se nanomaterials (start- and end-point micrographs are shown). Scale bar, 400 μm. (F) Quantitation of free wound area from wound-healing assay experiments. The data represent three separate experiments (*n* = 3) and are expressed as mean values ± SD. *p < 0.05, **p < 0.01, ***p < 0.005.

SEM micrographs of as-synthesized Se nanomaterials confirmed a succesful synthesis of two types of rod-like shaped (SeNR-1 and SeNR-2) and one type of spherical (SeNPs) nanoparticles (Fig. 1A). The morphology was further validated by AFM (Fig. 1B), and subsequent line scan analysis of AFM micrographs (Supplementary Fig. 2). It was found that both types of SeNRs exhibit comparable width (45 ± 4 nm for SeNR-1 and 45 ± 6 nm for SeNR-2) while differing in length (519 ± 87 nm for SeNR-1 and 1003 ± 194 nm for SeNR-2) and AR (∼11.5 for SeNR-1 and ∼22.3 for SeNR-2). Notably, SeNPs (with AR 1) exhibited HDD (38 ± 4 nm) comparable to the width of both SeNRs (Supplementary Fig. 3). All Se nanomaterials exhibited narrow size distribution with high dispersity in aqueous solution. In all Se nanomaterials, XPS analyses revealed relevant Se 3d peaks similar in shape with a split between Se 3d3/2 and Se 3d5/2 about 0.88 eV and with the areas close to a theoretical ratio (Fig. 2C). Binding energy of Se 3d5/2 about 55 eV is a typical feature of metallic Se [29]. Moreover, there is a weak broad peak around 58 eV that shows a loss feature typical for metals [30]. Further, both, XRF and organic elements composition analyses confirmed elemental composition of synthesized Se nanomaterials with no marked differences between the nanomaterials (Supplementary Fig. 4 and Supplementary Table 1). Further, the Raman spectra of Se nanomaterials demonstrate that all synthesized nanomaterials resembled trigonal bulk form of selenium^1^ (*t*-Se) (Supplementary Fig. 5), suggesting a well-developed crystalline structure that can promote high stability of Se nanomaterials. In addition, FT-IR analyses confirmed that chains of stabilizing agents (PVP or Na-CMC) are bound to the structure of Se nanomaterials, which is in line with the results of organic elements composition analyses. This was further validated by the C 1s and O 1s XPS analyses that confirmed the binding energies relevant to stabilizing agents (PVP or Na-CMC) present on the surface of Se nanomaterials (Supplementary Fig. 7).

In the next step, evaluated behavior (relative HDD changes) of Se nanomaterials in Ringer's solution mimicking the isotonic environment of bodily fluids, neutral buffer mimicking neutral intracellular environment and culture medium (DMEM supplemented with 10% FBS). Indeed, Fig. 1D shows that up to 24 h, Se nanomaterials are considerably stable. However, after 48 h, significant agglomeration of both types of SeNRs evolved particularly in neutral and Ringer's solutions. Interestingly, no significant agglomeration of any of Se nanomaterials was found during 72 h of incubation in DMEM. On the other hand, the incubation in DMEM (up to 48 h) led to some extent of dissolution of Se from particulate matter (Supplementary Fig. 8). This phenomenon was particularly evident for SeNPs (13.7% of dissolved Se after 48 h incubation), plausibly due to their higher specific surface area compared to SeNRs. Noteworthy, both types of SeNRs exhibited only neglectable Se dissolution, confirming their exceptional stability.

### 3.2 Evaluation of cytotoxic, antiproliferative and antimigratory activity

To delineate cytotoxic properties of Se nanomaterials *in vitro*, we first evaluated 24hIC_50_ values for non-malignant (HBL-100) and malignant (SH-SY5Y) cells. Fig. 2A demonstrates that the lowest 24hIC_50_ values were achieved for higher-AR SeNR-2 (27 μg/mL for SH-SY5Y and 70 μg/mL for HBL-100), followed by SeNPs (85 μg/mL for SH-SY5Y and 65 μg/mL for HBL-100). Trypan blue exclusion confirmed the trend of cytotoxicity and revealed that it is a dose-dependent phenomenon (Fig. 2B). Moreover, it was validated that Se nanomaterials did not interfere with XTT assay. Fig. 2C shows that SeNR-2 and SeNPs exhibited profound antiproliferative activity resulting in a significant decrease in amount of progeny colonies (Fig. 2D). Finally, we investigated possible inhibitory activity of Se nanomaterials to cellular migration. Fig. 2E demonstrates that in both cell lines, SeNR-2 and SeNPs are capable of inhibiting migration. In contrast, SeNR-1 exhibited only a limited ability to inhibit the closing of the artificial gap in cell monolayer (Fig. 2F). It is worth to note that the performed analyses are in good agreement and affirm the highest biological activity for higher-AR SeNR-2, followed by SeNPs and SeNR-1. Interestingly, the obtained data also indicate that SeNR-2 could possess some portion of a specificity to cancer cells.

### 3.3 Visualization of interactions between Se nanomaterials and cells *in vitro*

**Fig. 3:**
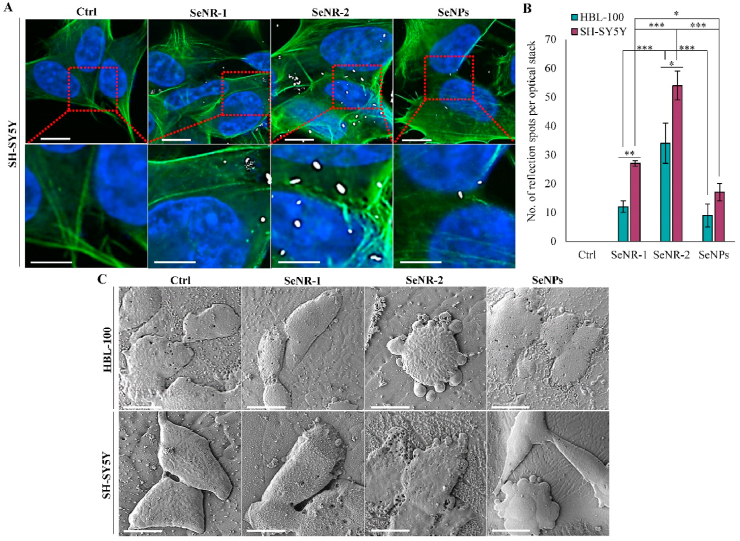
Evaluation of uptake Se nanomaterials and their effect on cell membranes and morphology. (A) Representative confocal reflection microscopy of uptake of Se nanomaterials by SH-SY5Y cells. Micrographs show maximum intensity Z-projections of cells stained with DAPI (nuclei, blue), FITC-labelled phalloidin (green) and reflection spots of Se nanomaterials (50 μg/mL, 3 h incubation, white). Scale bar, 10 μm (*upper panel*), 5 μm (*bottom panel*). (B) The bar graph shows the amount of Se nanomaterial reflection spots per optical stack. (C) Top-view cryo-FE-SEM micrographs of SH-SY5Y and HBL-100 cells treated with Se nanomaterials (50 μg/mL, 24 h). Scale bar, 10 μm. The data represent three separate experiments (*n* = 3) and are expressed as mean values ± SD. *p < 0.05, **p < 0.01, ***p < 0.005.

Next, we aimed to investigate if the Se nanomaterials are uptaken by cells and what is the subsequent effect of the exposure on cellular morphology. CRM analyses revealed that even after short incubation time (3 h), Se nanomaterials reside within the area demarcated by F-actin and are distributed within the intracellular space (Fig. 3A). The highest amount of reflection spots per optical stack was identified for SeNR-2 (34 for HBL-100 and 54 for SH-SY5Y) (Fig. 3B). However, it must be mentioned that the numbers obtained for SeNPs likely represent some portion of the intracellular agglomerates rather than individual SeNPs, which are below the CRM resolution [31]. Therefore, to validate the SeNPs uptake and intracellular distribution, additional analyses might be carried out. Follow-up cryo-FE-SEM analyses of cellular morphology revealed pronounced morphological hallmarks of ongoing apoptosis (cell shrinkage and membrane-bound apoptotic bodies) in particular due to the administration with higher-AR SeNR-2 (Fig. 3C).

### 3.4 Effect of Se nanomaterials on intracellular redox homeostasis and pDNA supercoiling

**Fig. 4:**
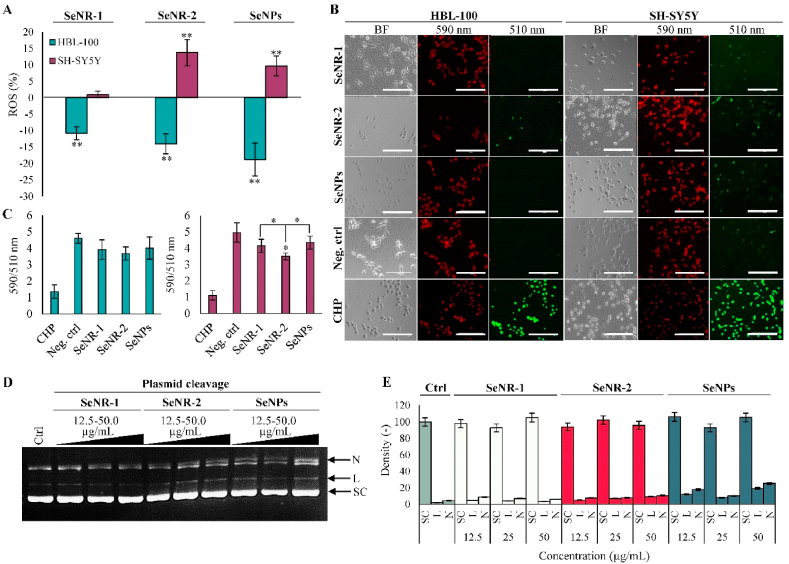
Examination of effect of Se nanomaterials on redox homeostasis. (A) Quantitation of intracellular ROS. (B) Representative micrographs of lipid peroxidation imaging. BF, brightfield. Scale bar, 200 μm. (C) Quantitation of fluorescence intensities of lipid peroxidation shown as a ratio of 590/510 nm. CHP, cumene hydroperoxide. (D) Representative EtBr-stained gel showing different conformations of pDNA in control and Se nanomaterials-exposed samples. (E) Densitometric quantitation of pDNA cleavage assay. SC, supercoiled, L, linear; N, nicked. The data represent three separate experiments (*n* = 3) and are expressed as mean values ± SD. *p < 0.05, **p < 0.01.

As a part of selenocysteine prosthetic group of selenoproteins, Se is an essential element crucial for cellular redox homeostasis. On the other hand, high concentrations of Se have been shown to induce extensive oxidative stress through deregulation of expression of antioxidant enzymes and lipid peroxidation [[32], [33], [34]]. Therefore, we further investigated how the morphology of Se nanomaterials affects their impact on intracellular redox homeostasis. We observed differential cell type-specific production of intracellular ROS (Fig. 4A). While in healthy (HBL-100) cells, all tested Se nanomaterials caused decline in ROS amount, in malignant (SH-SY5Y) cells, administration of SeNR-2 and SeNPs resulted in a significant induction of ROS production. Similar to ROS, SH-SY5Y cells exhibited enhanced susceptibility to Se nanomaterials-induced lipid peroxidation (Fig. 4B) with the highest peroxidation observed for higher-AR SeNR-2 (Fig. 4C). It is known that Se can cause DNA damage and fragmentation [35]. Hence, we further evaluated if Se nanomaterials interact with pDNA and affect its conformation. Fig. 4D demonstrates that upon incubation with pDNA, the highest rate of pDNA nicking was found for SeNPs (Fig. 4E). We anticipate that this phenomenon is closely related to the highest solubility of SeNPs (Supplementary Fig. 5) leading to the fastest release of reactive free Se willingly interacting with pDNA.

### 3.5 Intracellular fate of Se nanomaterials and their influence on cytoskeletal architecture

**Fig. 5:**
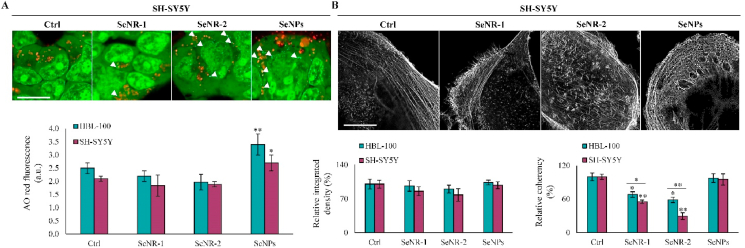
Se nanomaterials-induced lysosomal enrichment, LMD and cytoskeletal architecture reorganization. (A) Representative micrographs of AO relocation assay for visualization of lysosomes and LMD in SH-SY5Y cells. In control cells, lysosomes can be seen as red-orange puncta. In cells with LMD, lysosomes exhibit a shift to green color (white arrowheads). Scale bar, 20 μm. Bar graphs below indicate lysosomal enrichment (increase in red fluorescence) in both cell lines upon SeNPs administration. (B) Representative confocal micrographs of F-actin cytoskeleton (grey scale) in control and Se nanomaterials-administered SH-SY5Y cells. Scale bar, 5 μm. Bar graphs below show F-actin integrated density and coherency. *p < 0.05, **p < 0.01.

Intracellular ROS has been associated with LMD [36]. Therefore, we next investigated AO relocation in cells exposed to Se nanomaterials. As shown in Fig. 5A, in SH-SY5Y cells, only neglectable LMD was found for SeNR-1. In contrast, exposure to SeNR-2 and SeNPs resulted in some extent of LMD as evidenced by the reduction in red and enhancement of green fluorescence indicating the release of lysosomal content into the cytoplasm. Importantly, this observation is in line with the ability of SeNR-2 and SeNPs to trigger production of intracellular ROS (shown in Fig. 4A). In addition, we found that SeNPs caused significant (*p* < 0.05 for HBL-100 and *p* < 0.5 for SH-SY5Y) lysosomal enrichment in both cell lines, suggesting an efficient lysosomal accumulation of SeNPs, but not SeNRs, plausibly due to their size and spherical morphology. To further delineate the interaction between Se nanomaterials and cells, we also performed the morphometric evaluation of F-actin cytoskeletal architecture. As shown in Fig. 5B, the control and cells exposed to SeNPs displayed a number of well-organized thick bundles of F-actin. In contrast, both SeNRs caused disorganization and disruption of cytoskeletal network resulting in spike protrusions and a partial non-isotopic assembly of F-actin. In both cell lines, this phenomenon was reflected by slightly decreased F-actin integrated density and significant decrease in coherency, suggesting that nanorods-shaped Se nanomaterials display differential mode of interaction with cell membranes compared to SeNPs.

### 3.6 In vitro prediction of biocompatibility of Se nanomaterials

**Fig. 6:**
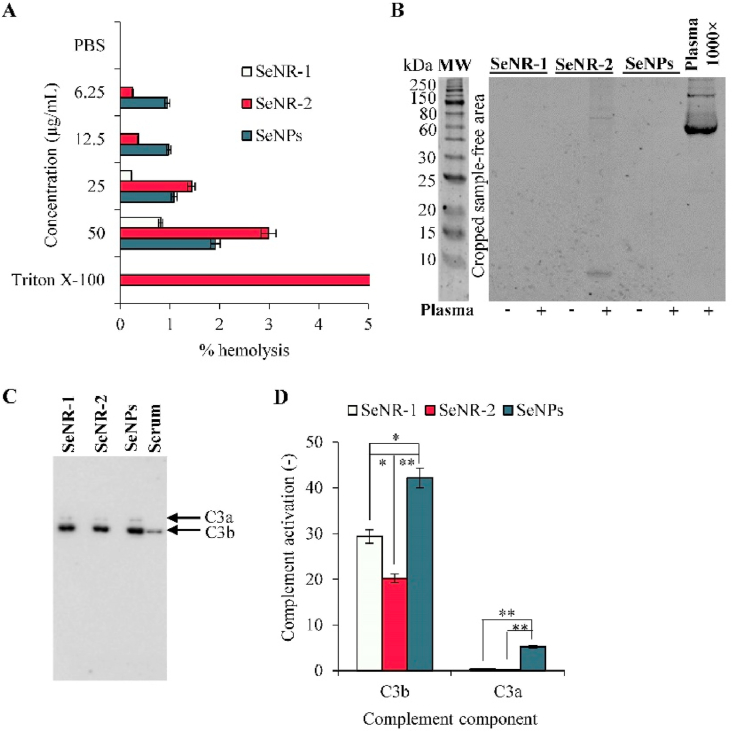
Investigation of *in vitro* biocompatibility of Se nanomaterials. (A) Hemocompatibility of Se nanomaterials assayed on human RBCs. (B) Gel showing eluted proteins constituting hard protein corona formed from proteins of human serum. (C) Immunoblot showing opsonization by complement components C3a and C3b. (D) Densitometric analysis of complement opsonization. *p < 0.05, **p < 0.01.

In the next step, we aimed on *in vitro* prediction of biocompatibility of Se nanomaterials in blood circulation. First, we investigated whether Se nanomaterials exhibit hemolytic activity in human RBCs. As shown in Fig. 6A, all tested Se nanomaterials caused only a neglectable disruption of RBCs with the highest observed hemolysis rate (<3%) found for SeNR-2. In addition, we found no (in the case of SeNR-1 and SeNPs) or only neglectable (SeNR-2) formation of hard protein corona on the surface of Se nanomaterials (Fig. 6B). We also examined the capability of Se nanomaterials to be opsonized by the complement component C3. It was found that irrespective of size and morphology, all tested Se nanomaterials bound opsonic complement fragment C3b (Fig. 6C). It is worth to note that the obtained data indicate that SeNPs exhibited the highest efficiency (*p* < 0.05) of opsonization and subsequent proteolytic cleavage to release C3a (Fig. 6D).

### 3.7 Biodistribution and short-term biocompatibility of Se nanomaterials

**Fig. 7:**
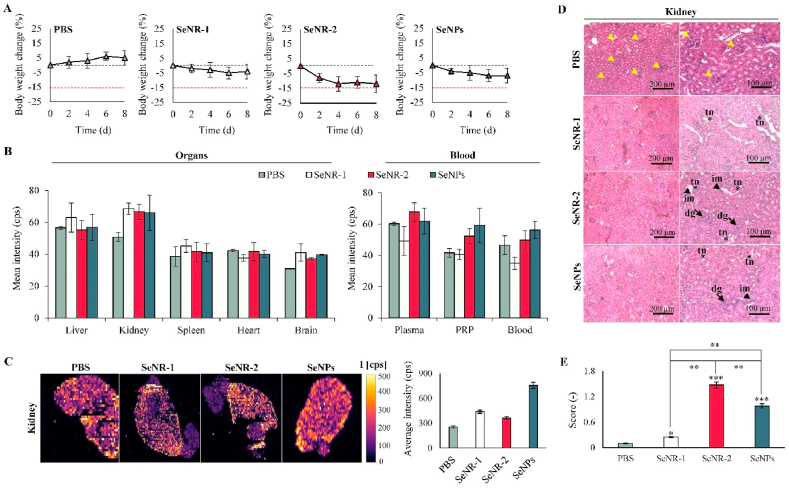
*In vivo* evaluation of biodistribution and biocompatibility of Se nanomaterials. (A) Relative changes in body weight of mice administered with PBS or Se nanomaterials. Black dashed line indicates zero value, red dashed line indicates a serious decrease in weight below 15%. (B) Quantitation of Se in selected organs (*left*) and components of blood (*right*). (C) LA-ICP-MS maps showing spatial distribution of Se in kidney of mice. Se intensities are shown in the bar graph (*right*). (D) Transversal H&E-stained tissue sections of kidney collected from mice administered to Se nanomaterials and PBS (control). Arrowheads indicate inflammatory infiltrates (im) in the cortex area, asterisks indicate tubular necrosis (tn) and arrows indicate onset of degeneration of glomeruli (dg). Control kidney sections show normal renal cortex and glomerular tufts (yellow arrowheads). (E) The bar graph showing the scoring of the histopathological changes. *p < 0.05, **p < 0.01, ***p < 0.005.

To examine the biological activity *in vivo*, BALB/c mice were administered intravenously with a single-dose application of Se nanomaterials. Generally, the treatments were well tolerated; neither death nor behavioral changes were observed during the 8 days observation period. Noteworthy, in contrast to control (PBS), Se nanomaterials caused some extent of body weight loss with the highest effect identified for SeNR-2 but not exceeding 15% loss of body weight from baseline weight indicating a serious decrease in body weight (Fig. 7A). ICP-MS analyses of total Se further revealed that after 8 days post-administration, slight increase of Se can be found in PRP from SeNR-2 and SeNPs-administered mice. Noteworthy, after 8 days the preferential site of bioaccumulation of Se nanomaterials, irrespective of size or morphology, were kidneys (Fig. 7B). Additional spatial determination of Se in kidney tissue sections confirmed bioaccumulation of Se with the highest accumulation for SeNPs (Fig. 7C). Despite comparable bioaccumulation of Se nanomaterials in kidney, histopathological examination revealed differential tissue damage manifested as inflammatory infiltrates in the cortex area, tubular necrosis and degeneration of glomeruli (Fig. 7D). The overall histopathological scoring summarized in Fig. 7E demonstrates that SeNR-2 caused the most extensive kidney tissue damage (*p* < 0.01 to control and *p* < 0.05 to SeNR-1 and SeNPs), which is in line with body weight analyses and higher-AR of SeNR-2. Interestingly, SeNPs administration resulted in a significantly (*p* < 0.05) larger kidney tissue damage compared to lower-AR SeNR-1. This phenomenon highlights that not only size/dimension/AR but also morphology is a crucial factor affecting the toxicological aspects of Se nanomaterials *in vivo*.

## 4 Discussion

Due to the widespread importance of Se for mammalian organism, Se nanoparticles belong among the most promising nanoscaled materials for biomedical and nutritional use. It has been already shown that by nanoformulating Se, it is possible to overcome the severe drawbacks (toxicity, poor retention, low efficiency, *etc*.) of organic or inorganic forms of Se [37]. Despite this fact, the available literature is inconsistent in the toxicological aspects of Se nanomaterials, and experimental data delineating the size/morphology relations with the biocompatibility of Se nanomaterials are lacking. Hence, the presented study represents the first comprehensive effort describing the mechanisms of cytotoxicity *in vitro*, and biocompatibility and biodistribution of Se nanomaterials differing in morphologies (nanorod-shaped *vs.* spherical) and sizes (comparable in one dimension, but varying in AR).

Despite the primary purpose of this study was not to study the cell-specific response to Se nanomaterials exposure, cytotoxicity screenings revealed that nanorod-shaped Se nanomaterials (in particular SeNR-2) exhibited higher cytotoxic potency in cancer cells (SH-SY5Y) compared to healthy epithelial cells (HBL-100). Although the mechanism beyond this phenomenon was not studied in detail, we anticipate that higher potency of SeNRs in cancer cells could be due to their increased membrane fluidity and decreased rigidity [38] allowing smoother penetration of SeNRs to the membranes. Considering the fact that nanorod-shaped particles have been shown to benefit from their morphology to align with bloodflow to increase the chance of convective delivery and penetrate tissues more efficient than spherical nanomaterials [39,40], the obtained data could serve as a basis for future studies focused on rational design of biomedical delivery vehicles with intrinsic selective cytotoxic properties.

In agreement with Wang et al. [41], we found that AR of nanorod-shaped nanomaterials plays a critical role in their nanotoxicity paradigm. Interestingly, throughout the whole study, we observed that spherical SeNPs exhibited lower cytotoxicity than high-AR SeNR-2, but higher than low-AR SeNR-1. This suggest that AR might indeed be a predictor of cytotoxicity of nanorod-shaped Se materials. However, to fully prove this phenomenon, additional studies with SeNRs exhibiting the same physico-chemical properties but a variety of ARs must be done.

It must be noted that whilst Na-CMC was used as a stabilizer for synthesis of SeNRs, SeNPs were stabilized using polyvinylpyrrolidone (PVP), therefore slightly differing in surface chemistry. However, since both, Na-CMC and PVP are highly biocompatible [42,43] and unbound stabilizers were removed by thorough washing, we anticipate that surface chemistry was not biasing the cytotoxicity data. It is worth to note that it was found that all synthesized Se nanomaterials resembled the same trigonal crystal phase of selenium^1^ (*t*-Se) and exhibited the same XPS peaks with binding energies typical for metallic Se. Thus, contribution of possible differences in inherent enzymomimetic activity due to differential crystal structure or chemical composition can be excluded, and differential cytotoxic behavior is more likely linked to the morphology and size of Se nanomaterials that are mechanistically affecting the interactions with cells.

The relation between cellular uptake/cytotoxicity of nanomaterials with their morphology and AR is still not well understood, and literature provides a plethora of contradictory results. While most of reports are in agreement that nanorod-shaped particles exhibit markedly higher internalization rates, most likely reminiscent of the advantage that rod-like bacteria utilize to enter non-phagocytic cells [[44], [45], [46]], contradictions can be found in the post-internalization biological effect. However, it must be noted that any comparative efforts are markedly complicated not only due to extensive physical, but also chemical variability. Our data are in line with the above-mentioned studies, and our CRM micrographs are suggestive of higher-AR SeNR-2 internalization.

To provide deeper insight into the biological activity of Se nanomaterials upon internalization, we aimed on determination of their deregulatory activity to intracellular redox homeostasis, which is a prominent causative mechanism of cytotoxicity of a broad spectrum of nanomaterials [47]. While some reports have shown antioxidant activity of Se nanomaterials [48], it is well-known that Se could cause serious oxidative damage [[32], [33], [34]]. It has been shown several times that Se nanoparticles can exhibit oxidase-like activity, and could therefore be able to produce H_2_O_2_ that can have deleterious effects on components of intracellular microenvironment [49,50]. Interestingly, our data indicate that ROS production by Se nanomaterials exposure is cell-specific. Whilst we found pronounced decrease in intracellular ROS in healthy epithelial cells, cancer cells exhibited significantly increased ROS formation upon administration with SeNR-2 and SeNPs. This could be explained by a generally higher basal ROS levels in cancer cells [51]. Further production of intracellular ROS by Se nanomaterials could led to the ROS accumulation above the threshold controlled by antioxidant pathways, leading to their disability to scavenge them.

We also show that Se nanomaterials can induce some extent of pDNA cleavage. Due to the fact that the highest pDNA cleavage was found for SeNPs with the highest dissolution rates, we anticipate that the damage to pDNA is rather caused by released Se (ability of Se to cause DNA damage is comprehensively reviewed in Ref. [52]) than by a direct interaction of Se nanomaterials with DNA.

Consistent with the recent report by Kirwale and coworkers [53], who demonstrated that SeNPs induce oxidative stress and follow-up formation of acidic lysosomes, we found that SeNPs, but not SeNRs caused significant lysosomal enrichment. This phenomenon could be explained by differential internalization mechanisms of Se nanomaterials related to their morphology. While SeNPs are most likely uptaken through endocytosis [54] enabling translocation of endosomal payload to lysosomes [55], we anticipate that nanorod-shaped SeNRs are rather internalized through transmembrane penetration that is common particularly for nanoparticles with larger dimensions and higher AR [56,57]. Noteworthy, this statement is further supported by significant alteration of the architecture of F-actin cytoskeletal network caused by SeNRs, but not by SeNPs.

Several previously published studies have shown that short-term, subchronic and acute toxicity of SeNPs in mice/rats is relatively low [37,58]. Indeed, our short-term experiments revealed that SeNR-1 and SeNPs could be well-tolerated in a long-term timeframe. In contrast, administration with SeNR-2 resulted in rapid weight loss observed already after 4 days post administration. Noteworthy, despite the differential effect on weight of exposed animals, we found that after termination of experiment; most of the Se nanomaterials reside in the kidneys, irrespective of size or morphology. This finding suggests that the studied Se nanomaterials tend to be cleared by renal clearance route, but all of them fell above the threshold required for kidney filtration, and are therefore accumulated until their degradation [59]. This phenomenon is frequently observed for nanoparticles with larger dimensions (reviewed in Ref. [60]). Importantly, despite the comparable bioaccumulation, Se nanomaterials exhibited differential ability to damage kidney tissue, which is in line with the presented *in vitro* data (SeNR-2>SeNPs > SeNR-1). Despite the study having some loopholes (*e.g*. short-time exposure, i.v. administration that could be complemented by i.p. and oral application) we provide a comprehensive insight into the biological behavior of Se nanomaterials. Our findings highlight that not only size but also morphology is a crucial factor deciding the biological activity of Se nanomaterials. While SeNR-2 and SeNPs could be interesting candidates for future rational development of delivery nanovehicles with intrinsic selective cytotoxicity to cancer cells with decreased rigidity and increased fluidity of membranes, SeNR-1 could serve as an interesting biocompatible platform with long on-site residence for delivery of bioactive substances to kidney tissue.

## 5 Conclusions

In summary, we provide a homogenous array of evidences showing that cytotoxicity and biocompatibility of Se nanomaterials are highly variable, and associates with their size and morphology. The highest cytotoxicity at the *in vitro* and *in vivo* level was found for nanorod-shaped Se particles with higher-AR (SeNR-2). We demonstrate that all tested Se nanomaterials are capable to be uptaken; however, the obtained data suggests that while SeNPs are endocytosed, Se nanorods directly enter the intracellular space by membrane penetration causing disorganization of cytoskeletal network. Accumulation of Se nanomaterials in intracellular region subsequently induced oxidative stress and LMD. Importantly, in contrast to Se nanorods, spherical Se nanoparticles were more prone to dissolution in physiological conditions leading to the highest rate of DNA nicking *in vitro*. *In vivo*, the preferential bioaccumulation site were kidneys, in which higher-AR SeNR-2 induced the highest rates of tissue damage. Taken together, the presented study shows that AR could be a descriptor of cytotoxicity of Se nanorods, but not spherical nanoparticles (always having AR 1.0) as these utilize different internalization routes and exhibit distinct stability in physiological fluids, which affect their biological activity. Aside from description of biological behavior of the Se nanomaterials differing in size and morphology, this study could offer new opportunities for a rational design of Se nanomaterials for biomedical or nutritional purposes.

## Credit author statement

Hana Stepankova: Investigation, Validation, Formal analysis; Hana Michalkova: Investigation, Methodology, Data curation; Zbynek Splichal: Investigation, Validation, Formal analysis; Lukas Richtera: Investigation, Data curation, Formal analysis, Validation; Pavel Svec: Investigation, Methodology, Data curation; Tomas Vaculovic: Investigation, Methodology, Data curation; Jan Pribyl: Investigation, Methodology, Data curation; Martin Kormunda: Investigation, Methodology, Data curation; Simona Rex: Conceptualization, Formal analysis, Data curation, Visualization Writing – Original Draft; Vojtech Adam: Conceptualization, Funding acquisition, Writing - Review & Editing; Zbynek Heger: Conceptualization, Writing - Review & Editing, Supervision, Project administration.

## Authors declaration of no conflict of interest

We wish to confirm that there are no known conflicts of interest associated with this publication and there has been no significant financial support for this work that could have influenced its outcome. We confirm that the manuscript has been read and approved by all named authors and that there are no other persons who satisfied the criteria for authorship but are not listed. We further confirm that the order of authors listed in the manuscript has been approved by all of us. We further confirm that any aspect of the work covered in this manuscript that has involved either experimental animals or human patients has been conducted with the ethical approval of all relevant bodies and that such approvals are acknowledged within the manuscript. We understand that the Corresponding Author is the sole contact for the Editorial process (including Editorial Manager and direct communications with the office). He is responsible for communicating with the other authors about progress, submissions of revisions and final approval of proofs. We confirm that we have provided a current, correct email address which is accessible by the Corresponding Author.
