# Supplementary material for: Unveiling the nanotoxicological aspects of Se nanomaterials differing in size and morphology
Source: Bioact Mater. 2022 Jun 25;20:489–500. doi: 10.1016/j.bioactmat.2022.06.014 (PMC9237951; doi:10.1016/j.bioactmat.2022.06.014)
Supplement: Multimedia component 1 [file mmc1.docx]

**Unveiling the Nanotoxicological Aspects of Se Nanomaterials Differing in Size and Morphology**

Hana Stepankova^1^, Hana Michalkova^1,2^, Zbynek Splichal^1,2^, Lukas Richtera^1,2^, Pavel Svec^1,2^, Tomas Vaculovic^3^, Jan Pribyl^4^, Martin Kormunda^5^, Simona Rex^1,2^, Vojtech Adam^1,2^, Zbynek Heger^1,2*^

*^1^Department of Chemistry and Biochemistry, Mendel University in Brno, Zemedelska 1, Brno CZ-613 00, Czechia*

*^2^Central European Institute of Technology, Brno University of Technology, Purkynova 123, Brno CZ-612 00, Czechia*

*^3^Department of Chemistry, Masaryk University, Kamenice 5, Brno CZ-625 00, Czechia*

*^4^Central European Institute of Technology, Masaryk University, Kamenice 5, Brno CZ-625 00, Czechia*

*^5^Department of Physics, Faculty of Science, J. E. Purkyne University, Pasteurova 1, Usti nad Labem CZ-400 96, Czechia*

Corresponding Author

*Zbynek Heger, Mendel University in Brno, Zemedelska 1, Brno CZ-613 00, Czechia; E-mail: [zbynek.heger@mendelu.cz](mailto:zbynek.heger@mendelu.cz); phone: +420-5-4513-3350; fax: +425-5-4521-2044

**Experimental section**

Energy dispersive X-ray fluorescence

An elemental analyzer SPECTRO XEPOS energy dispersive X-ray fluorescence (ED-XRF) spectrometer (SPECTRO Analytical Instruments GmbH, Kleve, Germany) equipped with a 10 mm^2^ Si-Drift detector with Peltier cooling and a 75 µm Be side window was employed. The instrument uses a Pd-target end window tube at a maximum power of 50 W and a maximum voltage of 50 kV. Spectral resolution of the instrument (FWHM) is < 170 eV for Mn Kα (measured under input count rate 10,000 pulses). SPECTRO XEPOS was operated and data were evaluated by means of the software Spectro X-Lab Pro, Version 2.5. For light elements excitation (Mg-V, tube voltage 24.82 kV, tube current 1.00 mA, measurement duration 300 s, impulse rate ≤ 870 cps, relative dead time ≤ 1.4 %, peak time 2 µs, gain 12.5 eV channel^−1^, zero peak rate 5,000 cps) highly oriented pyrolithic graphite (HOPG) crystal target was used. For heavier elements determination Mo secondary target (Cr-Y, Hf-U, tube voltage 44.70 kV, tube current 0.55 mA, measurement duration 300 s, impulse rate ≤ 1,900 cps, relative dead time ≤ 1.7 %, peak time 2 µs, gain 25.0 eV channel^−1^, zero peak rate 5,000 cps) and Al_2_O_3_ polarization target (Zr-Ce, tube voltage 49.16 kV, tube current 0.50 mA, measurement duration 500 s, impulse rate ≤ 100 cps, relative dead time ≤ 0.6 %, peak time 2 µs, gain 50.0 eV channel^−1^, zero peak rate 5,000 cps) was used. Solid sample (thin film containing few mg of Se nanomaterials after evaporation of water from the dispersion) were measured directly in sample cup (32 mm in diameter) on polypropylene X-Ray thin-film TF-240, 4 µm (FluXana, Bedburg-Hau, Germany) and measured in vacuum using the Turboquant method. To prevent sample loss, Se nanomaterials were placed between two polypropylene foils.

*Analysis of organic elements composition*

Elemental analysis was performed using Flash 2000 Organic Elemental Analyzer (Thermo Fisher Scientific Inc., Waltham, MA, USA). Carbon, hydrogen, nitrogen and sulfur were determined simultaneously in one measurement, oxygen was determined in a separate analysis. Two independent samples were measured for each determination and the average values are presented. Water dispersions of Se nanomaterials were centrifuged, obtained solids were washed twice with absolute ethanol followed by centrifugation and dried at 85°C to remove all of the solvent.

*Raman spectroscopy*

Raman spectra were recorded on dispersive inVia Reflex Raman microscope (Renishaw, Cheltenham, UK) with integrated Leica microscope DM2700 (Leica, Wetzlar, Germany) and controlled by Wire 5.2 software (Renishaw). Diode laser with an excitation wavelenght 633 nm and 17 mW power was used. Spectra were collected over the approximate range of 100–3,200 cm^−1^ using microscope immersion objective 50× and a power setting of 100 %. Each spectrum was acquired by merging 32 scans.

*Fourier-transform infrared spectroscopy*

Fourier-Transform Infrared (FT-IR) spectra were collected using INVENIO-R FT-IR Spectrometer equipped with a single-reflection diamond attenuated total reflectance (ATR) accessory - A225/Q Platinum ATR module (Bruker Optic Inc., Billerica, MA, USA). Small amount of a solid sample was put on crystal and fixed load was applied to the sample to ensure full contact with the diamond ATR. Water dispersions of Se nanomaterials were centrifuged and separated into solid products and residual supernatants. Se nanomaterials were put on a crystal in a form of concentrated water dispersions and after drying using a small electrical fan, spectra were collected. 2 mL volumes of residual supernatants were then concentrated into a minimal volumes to obtain approx. 40×-concentrated samples which were then measured in the same way like selenium products. Before each measurement background spectra were collected. Spectra were recorded at 25°C from 4,000 to 400 cm^−1^ at a resolution of 2 cm^−1^. Each spectrum was acquired by merging 128 interferograms. Bruker OPUS software was used for IR spectra recording and processing.

**Results**


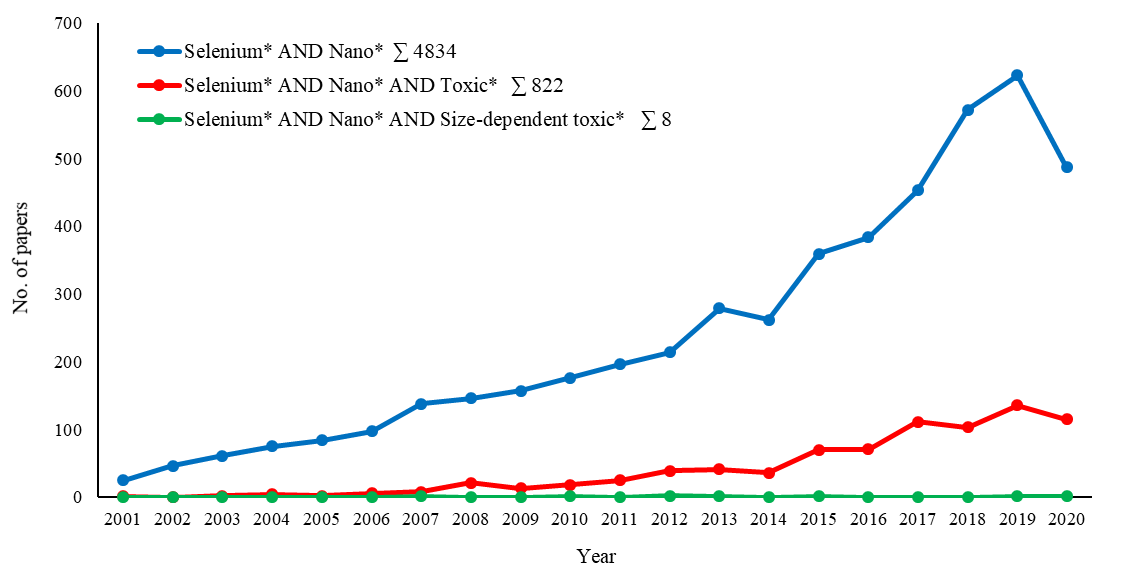


**Supplementary figure 1**: Number of papers available in Web of Science (Thomson Reuters) under the terms Selenium* AND Nano*, Selenium* AND Nano* AND Toxic* and Selenium* AND Nano* and Size-dependent toxic*. Total sums of papers are shown as well. Search was performed on September 10^th^, 2020.


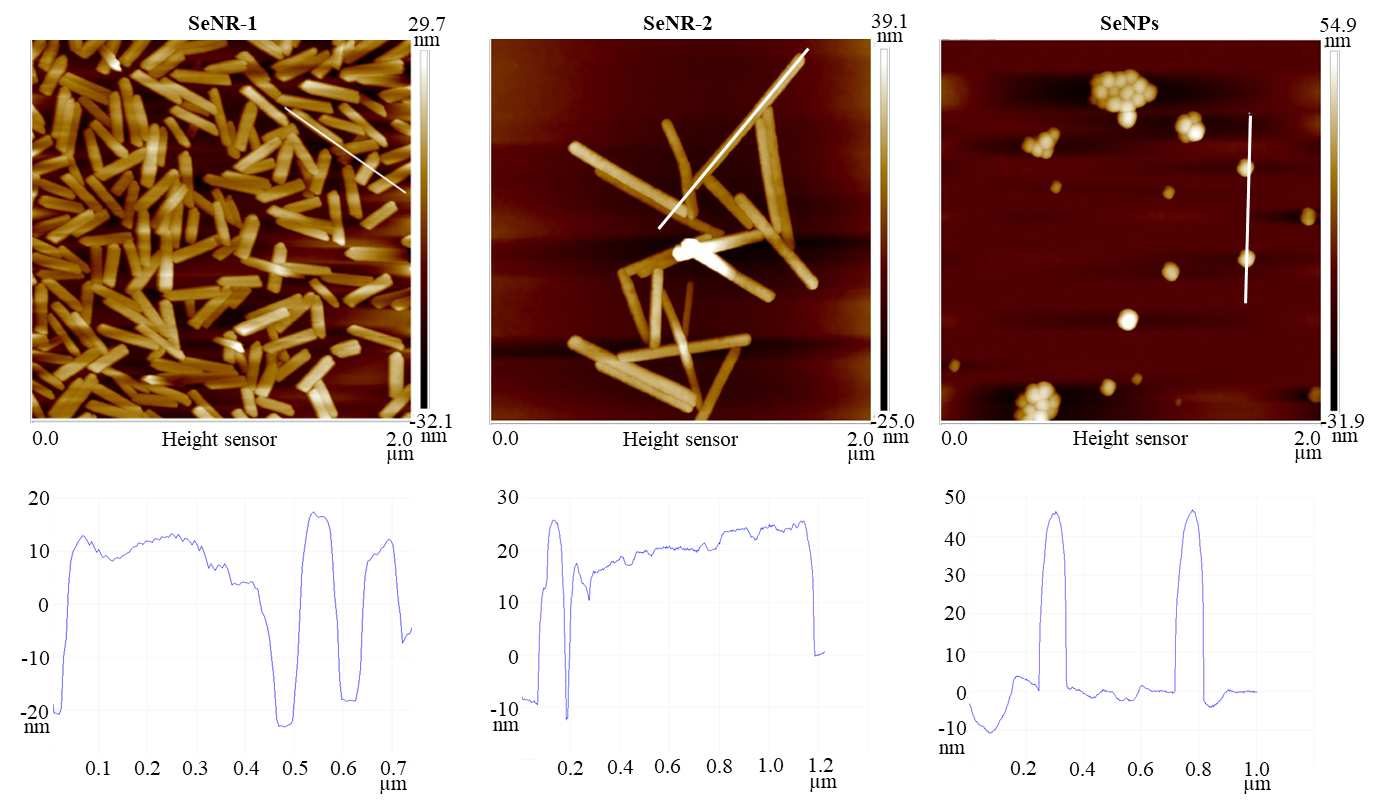


**Supplementary figure 2:** 2D AFM micrographs with line scans showing the size of Se nanomaterials.


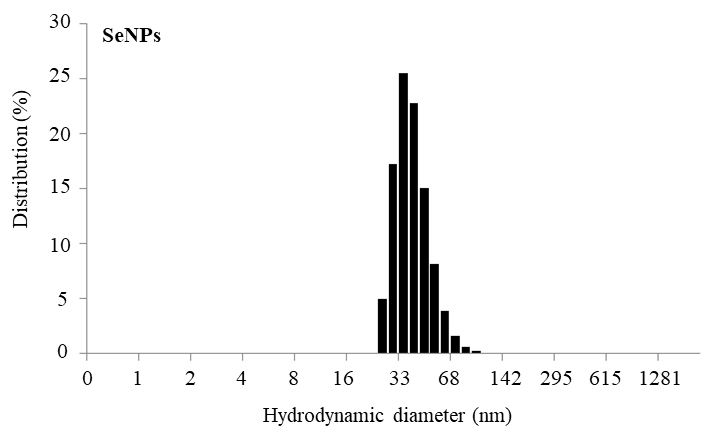


**Supplementary figure 3:** DLS histogram showing HDD of SeNPs.


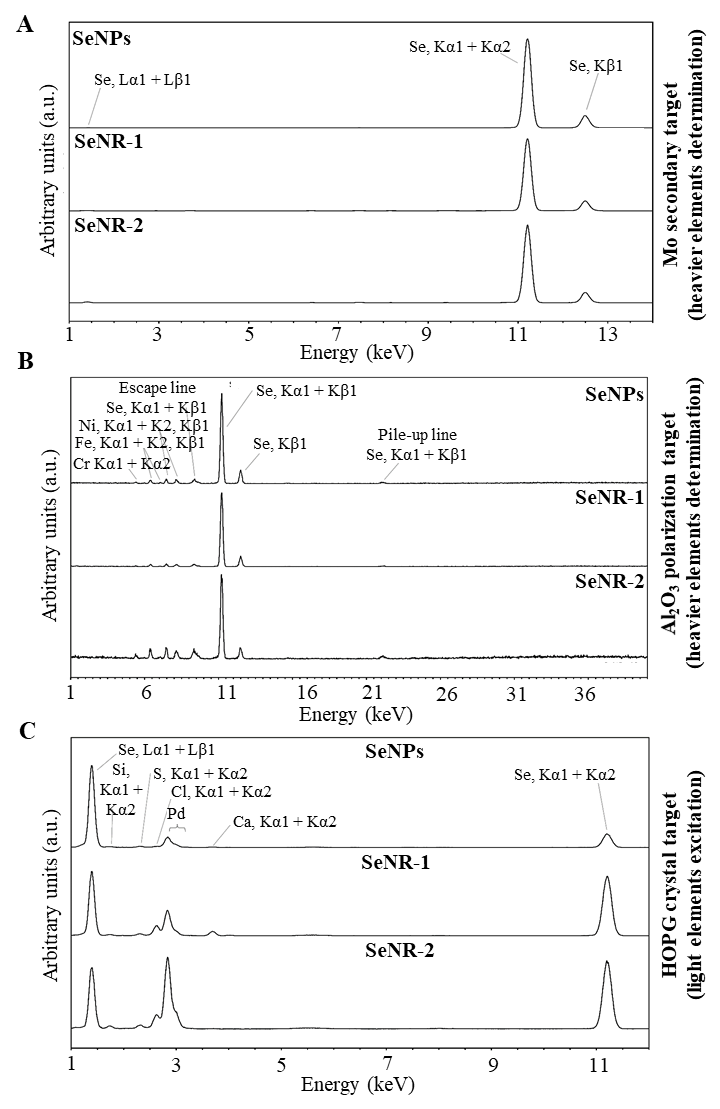


**Supplementary figure 4:** XRF spectra confirming elemental composition of prepared Se nanomaterials. (**A**) Measurements with Mo secondary target, (**B**) measurements with Al_2_O_3_ polarization target and (**C**) measurements with HOPG crystal target.

**Supplementary table 1:** CHNSO elemental analysis of Se nanomaterials

| **Sample** | **C**  **(%)** | **H**  **(%)** | **N**  **(%)** | **S**  **(%)** | **O**  **(%)** |
| --- | --- | --- | --- | --- | --- |
| **SeNPs** | 0.70±0.03 | 0.09±0.00 | 0.62±0.18 | 0.04±0.04 | 0.61±0.23 |
|  |  |  |  |  |  |
| **SeNR-1** | 2.74±0.10 | 0.33±0.03 | 0.11±0.04 | 0.00±0.00 | 2.57±0.09 |
|  |  |  |  |  |  |
| **SeNR-2** | 2.20±0.08 | 0.30±0.02 | 0.04±0.02 | 0.00±0.00 | 1.49±0.02 |
|  |  |  |  |  |  |


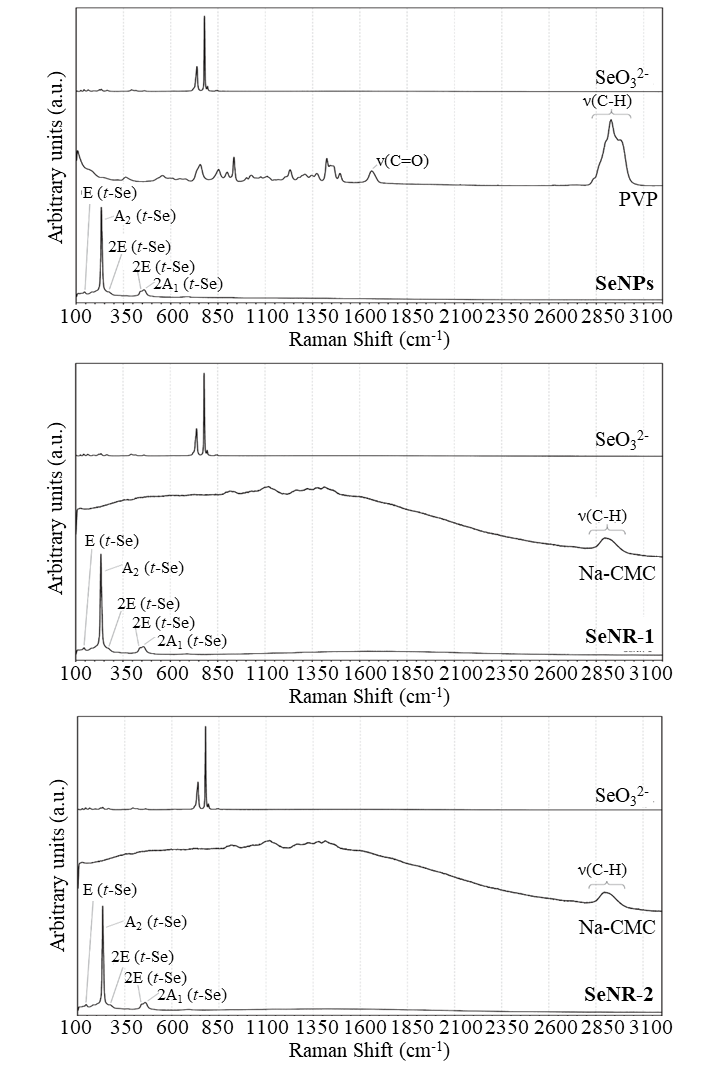


**Supplementary figure 5:** Raman spectra of Se nanomaterials, coating agents (PVP and Na-CMC) and SeO_3_^2-^ (in a form of sodium selenite). For all Se nanomaterials, Raman spectra consist only of peaks belonging to trigonal form of selenium^1^ (*t*-Se) with the most prominent peak at 233 cm^-1^.


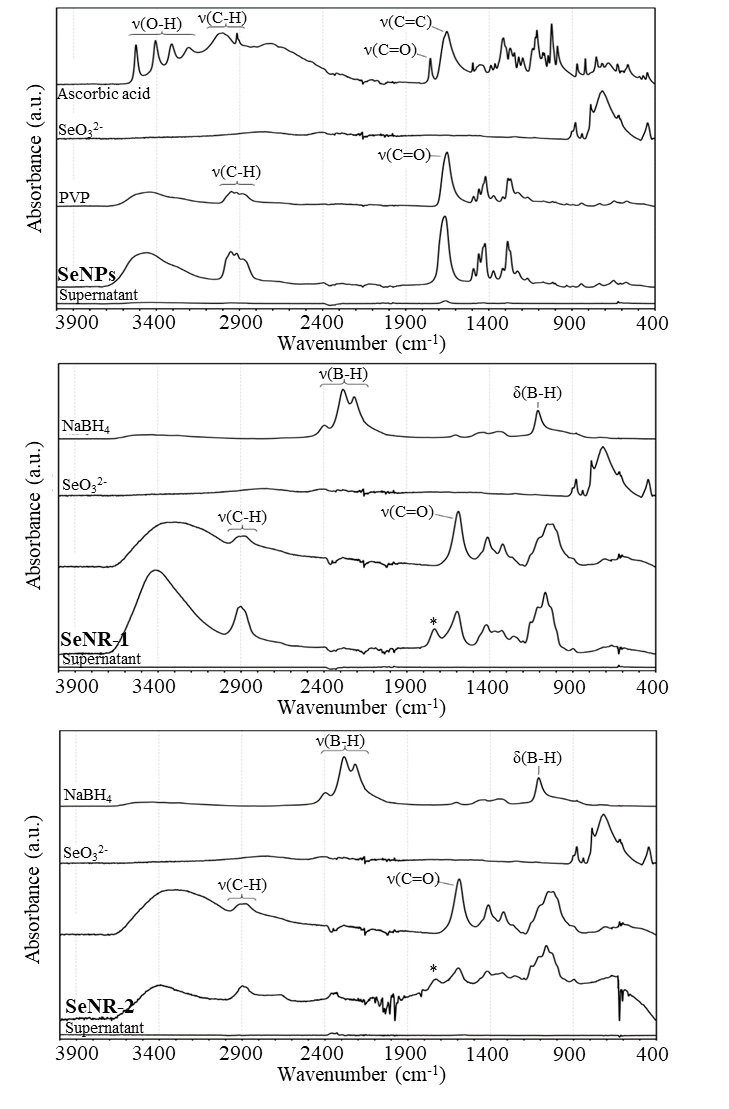


**Supplementary figure 6:** FT-IR spectra of Se nanomaterials including residual approx. 40× concentrated supernatant, PVP, Na-CMC, SeO_3_^2-^ (in a form of sodium selenite) and reducing agents (ascorbic acid or NaBH_4_).


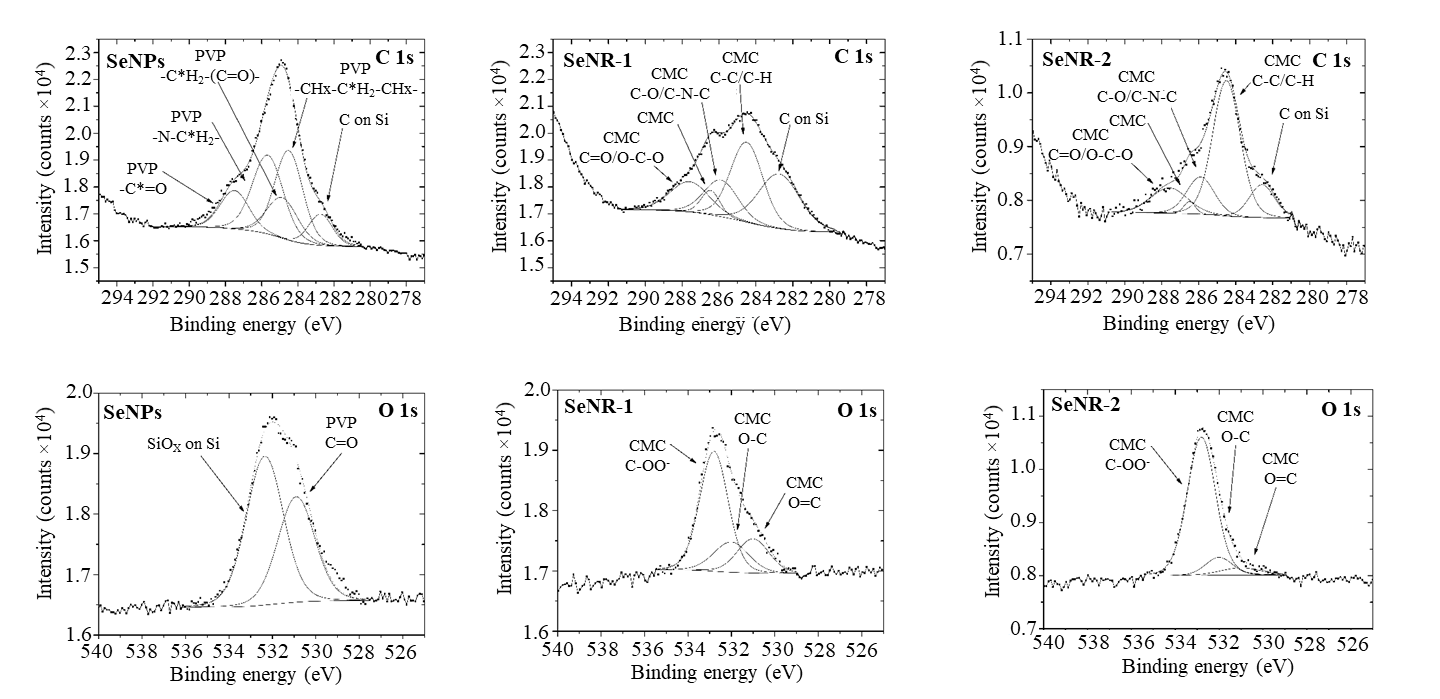


**Supplementary figure 7:** XPS spectra showing C 1s and O 1s peaks of synthesized Se nanomaterials.

**
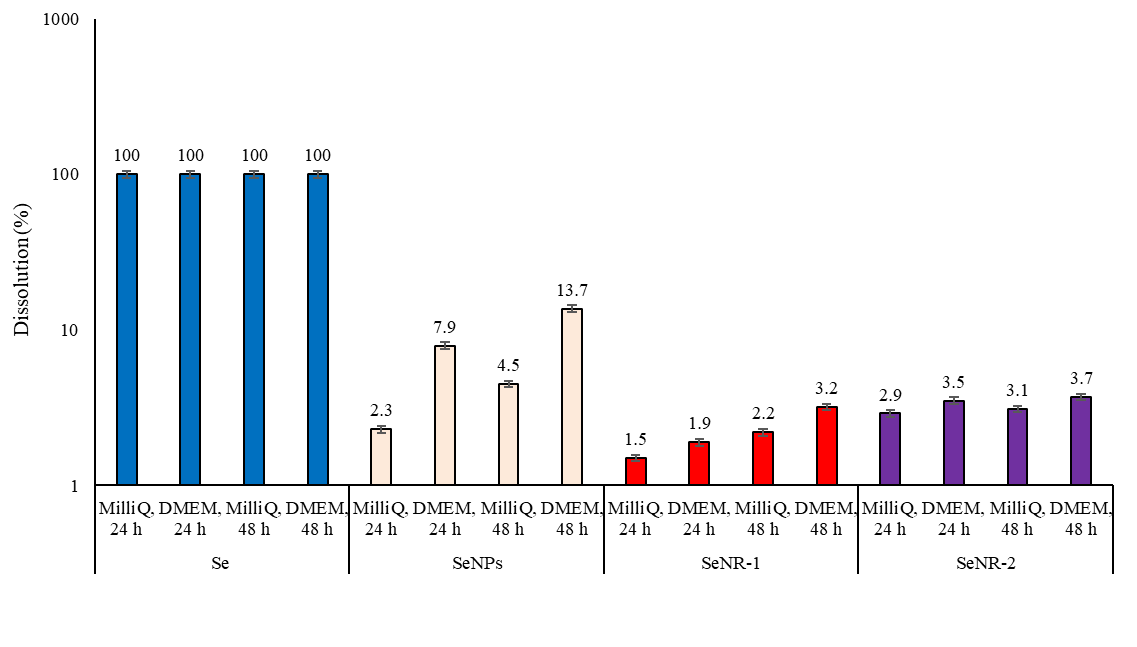
Supplementary figure 7:** Dissolution (%) of Se nanomaterials in MilliQ water and cell culture medium (DMEM).
